# Research of intelligent reasoning system of *Arabidopsis thaliana* phenotype based on automated multi-task machine learning

**DOI:** 10.3389/fpls.2023.1048016

**Published:** 2023-02-14

**Authors:** Peisen Yuan, Shuning Xu, Zhaoyu Zhai, Huanliang Xu

**Affiliations:** College of Artificial Intelligence, Nanjing Agricultural University, Nanjing, China

**Keywords:** plant phenotype reasoning, multi-task learning, automated machine learning, *Arabidopsis thaliana*, cloud deployment

## Abstract

Traditional machine learning in plant phenotyping research requires the assistance of professional data scientists and domain experts to adjust the structure and hy-perparameters tuning of neural network models with much human intervention, making the model training and deployment ineffective. In this paper, the automated machine learning method is researched to construct a multi-task learning model for *Arabidopsis thaliana* genotype classification, leaf number, and leaf area regression tasks. The experimental results show that the genotype classification task’s accuracy and recall achieved 98.78%, precision reached 98.83%, and classification *F*_1_ value reached 98.79%, as well as the *R* ^2^ of leaf number regression task and leaf area regression task reached 0.9925 and 0.9997 respectively. The experimental results demonstrated that the multi-task automated machine learning model can combine the benefits of multi-task learning and automated machine learning, which achieved more bias information from related tasks and improved the overall classification and prediction effect. Additionally, the model can be created automatically and has a high degree of generalization for better phenotype reasoning. In addition, the trained model and system can be deployed on cloud platforms for convenient application.

## 1 Introduction

Plant phenotypes are recognizable morphological, physiological, and biochemical characteristics and attributes that result in part or entirely from the interaction of genes with the environment (Dobrescu et al., 2020; Cheng et al., 2021; Saric et al., 2022), which is widely used in ecological protection (Carvalho et al., 2021), plant breeding (van Dijk et al., 2021) and so on. Currently, machine learning has rapidly evolved and is now widely applied in science in general and in plant genotyping and phenotyping (Ubbens and Stavness, 2017; van Dijk et al., 2021). Different phenotypic qualities of plants are connected with one another, and this gives us the hints to leverage the benefits of multi-task learning to enhance the effectiveness of individual learning activity. In order to improve the classification and prediction performance of numerous related tasks, multi-task learning for the same plant enables better reasoning of the link between various phenotypic variables as well as training with less data and annotated information.

The construction of multi-task learning models requires a lot of manual time to tune the hyperparameters of the model for making the model have a high performance (Zhang et al., 2019; Vandenhende et al., 2022).

Therefore, there are limitations in human thinking to consider the model structure and parameters in all possible cases.

At the present time, machine learning has become an essential part of daily applications (van Dijk et al., 2021), however, building well-performing machine learning models still requires the help of data scientists and domain experts. To solve this problem, Automated Machine Learning (AutoML for short) (He et al., 2021) was proposed and researched. AutoML automates the process of constructing network structure, adjusting network structure, adjusting hyperparameters, and model evaluation (Truong et al., 2019; Xue et al., 2021) through its own set of algorithms, which turns the original structure adjustment and parameter tuning into structured and orderly adjustment through the well-designed algorithms, which lowers the threshold of machine learning and shortens the whole modeling process. Using automated machine learning methods enables deep learning techniques to be applied to more fields in a simpler way to build better network models for machine learning tasks with high accuracy. AutoML brings a way for researchers without AI knowledge and the help of machine learning experts to build their AI system (Zöller and Huber, 2021).

Based on the above pros and cons, we propose the AutoML to build a multi-task learning model for *Arabidopsis thaliana* phenotype reasoning, which can combine the advantages of both approaches, and take into account both the correlation between multiple phenotypic variables and the diverse model structures and parameter pairings. For multi-task learning, the use of an automated machine learning method to construct models provides a viable new approach for subsequent research on other multitasks. And for AutoML, applying the knowledge of multitask learning allows for better finding the network suitable for each task by taking into account the correlation between tasks when searching for neural network architectures.

Currently, Zhou et al. (2021) introduced a deep learning-based maize image analysis software that can automatically solve a variety of image-based maize phenotyping tasks, such as internal length, stem diameter, and leaf count, for high-throughput plant phenotyping. Similarly, P. Hüther et al. (Hüther et al., 2020). analyze the phenotype of *Arabidopsis thaliana* using transfer learning by centering our pipeline around the well-established deep-learning model DeepLabV3+ for batch automated plant leaf state analysis, and no automated generation of the model was implemented.

This paper focuses on automated machine learning methods for multi-task learning models. Taking *Arabidopsis thaliana* as an example, three tasks concerning the processing and analysis of plant phenotypic characteristics were finally realized: 1)infer the genotype of *Arabidopsis thaliana*; 2) predict the total number of leaves in *Arabidopsis thaliana*; and 3)predict the leaf area of *Arabidopsis thaliana*. For the above analysis tasks of *Arabidopsis thaliana* dataset, AutoML based multi-task models are researched and constructed for the three tasks mentioned above for training, and the different metrics of each model are compared to produce the best classification and regression results.

The main contribution of this paper is the use of an automated machine learning approach that automatically adjusts hyperparameters as well as model structure as a way to construct a multi-task learning model for *Arabidopsis thaliana* phenotype reasoning tasks. And the experiment results show that it has better classification and regression results compared to previous state-of-the-art models.

The rest of this paper is organized as follows. Section 2 introduces the relevant principles and workflow of multi-task AutoML. The details of the multi-task AutoML model used in this paper are explained in Section 3. Experiment and comparison of the proposed method for *Arabidopsis thaliana* phenotype multi-task reasoning are presented in Section 4. System implementation and deployment are presented in Section 5. Finally, Section 6 draws conclusions and provides an in-depth analysis and an outlook on future work.

## 2 Related works

Multi-Task Learning (MTL for short) has been proposed with the intention of leveraging the useful information contained in multiple related tasks to help improve the generalization performance of all the tasks (Zhang and Yang, 2022). While the phenotypic traits of plants are correlated to some extent, using multi-task learning, the network can be trained with less data and less labeled information to achieve better classification and prediction results for multiple related tasks.

Among the two basic frameworks for multi-task learning, soft parameter sharing does not make any assumptions about task relevance, but the number of required parameters is large. In contrast, hard parameter sharing is mostly applied to networks with strong task relevance. For the study of *Arabidopsis thaliana* phenotypes, there are strong correlations among phenotypes, and thus the hard parameter sharing framework is mostly used to build relevant models. For example, the first application of multi-task learning to plant phenotypes was proposed by Pound et al. (2017) with the ability to both detect and count the number of wheat ears and to classify the presence of wheat awns, and Dobrescu et al. (2020) present a hard parameter sharing framework of multi-task learning for plant phenotyping to infer leaf count, projected leaf area, and genotype classification.

With the development of multi-task learning, the simple hard parameter sharing model can no longer satisfy the needs of *Arabidopsis thaliana* phenotype multi-task reasoning applications, and people start to add different strategies to the hard parameter sharing framework to obtain higher performance. Jiang et al. (2021) incorporates a migration learning approach in a hard parameter sharing framework to achieve simultaneous recognition of leaf diseases in rice and wheat. Keceli et al. (2022) combined CNN features and transfer features to construct a multi-input multi-task learning model to improve the efficiency of plant type and disease detection.

Automated Machine Learning is the process of automating the end-to-end process of applying machine learning to real-world problems, enabling models to automatically learn appropriate parameters and models without human intervention.

Currently, the open source AutoML frameworks include Auto-sklearn (Feurer et al., 2015), TPOT (Olson et al., 2016), Auto-Keras (Jin et al., 2019), H2O (LeDell and Poirier, 2020), etc. Auto-sklearn (Feurer et al., 2015) and H2O (LeDell and Poirier, 2020) are mainly oriented to traditional machine learning for automatic modeling. TPOT (Olson et al., 2016) mainly applies genetic algorithms for feature and model selection. Auto-Keras (Jin et al., 2019) is a Keras- based AutoML system that can achieve the powerful function of neural architecture search with only a few lines of code and is easy to get started and use.

Nowadays, More and more advanced methods are being applied to AutoML to improve the performance of the models. Wong et al. (2018) proposed Transfer Neural AutoML, which reduces the computational cost of neural AutoML by migration learning. Xue et al. (2019) proposed a migratable AutoML method that uses previously trained models to speed up the search process for new tasks and datasets, accelerating the overall search time for multiple datasets with negligible accuracy loss. Ferreira et al. (2021) conducted a comparative study of hundreds of computational experiments based on a total of three scenarios: general-purpose machine learning, deep learning, and XGBoost, with GML achieving the best prediction results and the GML AutoML tool obtaining the most competitive results, while confirming the potential of the general-purpose AutoML tool to fully automate machine learning algorithm selection and tuning. Zöller et al. (2022) proposed an XAutoML for interpreting arbitrary AutoML optimization processes and ML pipelines constructed by AutoML. And the framework we use is optimized for AutoML mainly in the Neural Architecture Search part.

Neural Architecture Search (NAS) (Elsken et al., 2019) aims at automatically designing well-performing neural network architectures for specific target tasks, which requires huge computational resources. Ying et al. (2019) introduced NAS-Bench-101 to ameliorate these problems. And Dong et al (Dong and Yang, 2020). proposed NAS-Bench-201 with a fixed search space, which provides a unified benchmark for almost all the latest NAS algorithms and is an extension of NAS-Bench-101. To overcome the efficiency challenges of simple weight sharing in NAS, Shen et al. (2022) introduce DASH, a differentiable NAS algorithm that achieves better asymptotic complexity and up to 10 times faster search time in practice. Luo et al. (2020) proposed SemiNAS, a semi-supervised NAS approach that uses a trained accuracy predictor to predict the accuracy of a large number of architectures, reducing computational cost and achieving higher accuracy at the same computational cost with the same accuracy guarantee, e.g., it achieves 94.02% test accuracy on NASBench-101, using the same number of architectures outperformed all baselines. Xue et al. (Xue and Qin, 2022). proposed a partial channel connection based on channel attention for differentiable neural architecture search. Auto-Keras (Jin et al., 2019) uses an efficient neural architecture search method with network morphism, combined with Bayesian optimization, which makes the search space exploration more efficient and has better performance for the current optimal baseline model.

In the field of plant phenotype research, Koh et al. (2021) investigated the application of AutoML in image-based plant phenotyping, comparing the performance of the open source AutoML framework Auto-Keras with migration learning using a convolutional neural network architecture. In the classification task, migration learning with Xception (Chollet, 2017) and DenseNet-201 (Huang et al., 2017) achieved the best classification accuracy of 93.20%, while Auto-Keras achieves 92.40% accuracy. With similar accuracy, Auto-Keras speeds up the model’s inference time by a factor of 40 and has great potential for enhancing plant phenotyping capabilities applicable to crop breeding and precision agriculture.

In summary, we proposed an intelligent reasoning system for *Arabidopsis thaliana* phenotype based on automated Multi-task machine learning with Auto-Keras (Jin et al., 2019), which can take the both advantages of AutoML and multi-task learning.

## 3 Intelligent reasoning of *Arabidopsis thaliana* phenotype based on multi-task automated machine learning

### 3.1 Problem statement

In AutoML, model generation and evaluation are done by neural network architecture search. As the backbone of deep AutoML, Neural Architecture Search(NAS for short) (Xue and Qin, 2022) can define and optimize the neural network architectures and tune hyperparameters automatically, which enables people with little expertise and knowledge to perform machine learning tasks for obtaining highly accurate, and even discover unproposed network architectures for some specific tasks.

**Figure 1:**
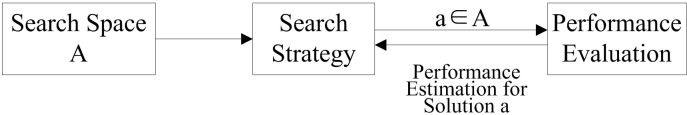
Basic processing procedure of neural architecture search for auto machine learning.

The basic process of NAS is shown in **Figure 1**. First, a specific structure a is selected from the predefined search space A according to the search strategy, and the specific structure is evaluated by the performance evaluation module, which returns the performance estimate to the search strategy and guides the next structure selection, and so on until a model a∗ satisfying the predefined performance requirements is produced as the output of the problem.

The optimization strategy used by NAS to obtain the optimal network configuration is shown in Equation 1.


(1) $$\begin{array}{l} a*=\text{ arg max O (Λ}(a,\;d_{t}),\;d_{\text{v}}\;=\text{ arg max }f\;(a)\\ \;a\in A\;a\in A \end{array}$$

where O represents the target function for training network structure parameters, *d_t_* represents training data, and *d*_v_ represents validation data.

Among them, the calculation formula of Λ(*a*, *d*) is shown in Equation 2.


(2) $$\begin{array}{l} \Lambda (a,\;d)\;=\text{ arg min L(}m_{a}{,}_{\text{θ, }}d_{t}\text{) + R(θ)}\\ \;m_{a,\text{θ}}\in M_{a} \end{array}$$

where, *M* is the model space, L represents the loss parameter used for the training network, θ represents the network parameter, and R represents the loss function part used for regularization.

### 3.2 Workflow of *Arabidopsis thaliana* phenotype analysis based on automated multitask machine learning

**Figure 2:**
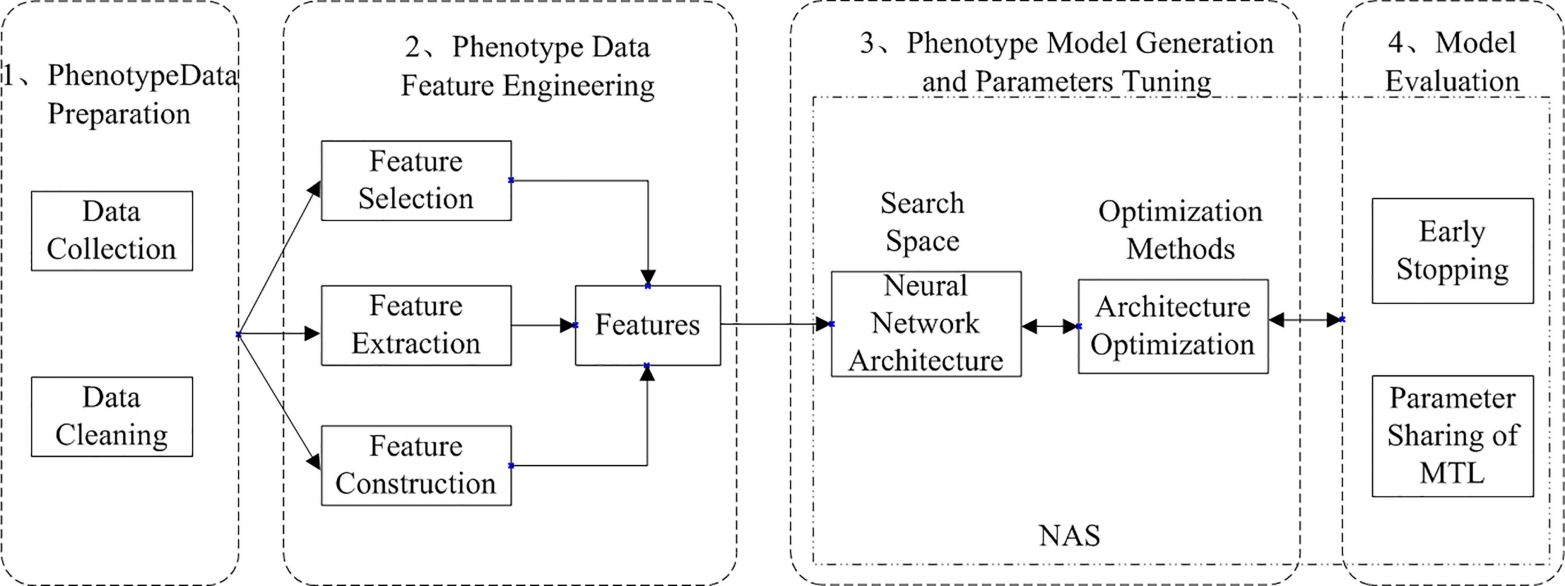
Workflow of *Arabidopsis thaliana* phenotype analysis based on automated multi-task machine learning (He et al., 2021).

AutoML can be divided into two types: traditional AutoML and deep AutoML. Traditional AutoML combines the three steps of feature engineering, model selection, and optimization algorithm selection into a single pipeline for automatic learning. Deep AutoML uses neural architecture search (Elsken et al., 2019) to optimize the three problems and thus learn the optimal network structure automatically. The deep AutoML for neural network modeling in deep learning compose of four processes: data preparation, feature engineering, model generation and evaluation, and the workflow of *Arabidopsis thaliana* phenotype with auto multi-task reasoning is shown in **Figure 2**.

#### 3.2.1 Data preparation

The preparation of *Arabidopsis thaliana* phenotype data mainly includes data collection and data cleaning.

1. Data collection consists mainly of data collection, data tagging, and data improvement (Roh et al., 2019), which tunes the completed raw data into the storage systems.

Data collection includes the following two steps. The main purpose is to convert the Ara2013-Canon dataset (Minervini et al., 2016) to Visual Object Class (VOC) format to obtain direct information about leaf area.

**Figure 3:**
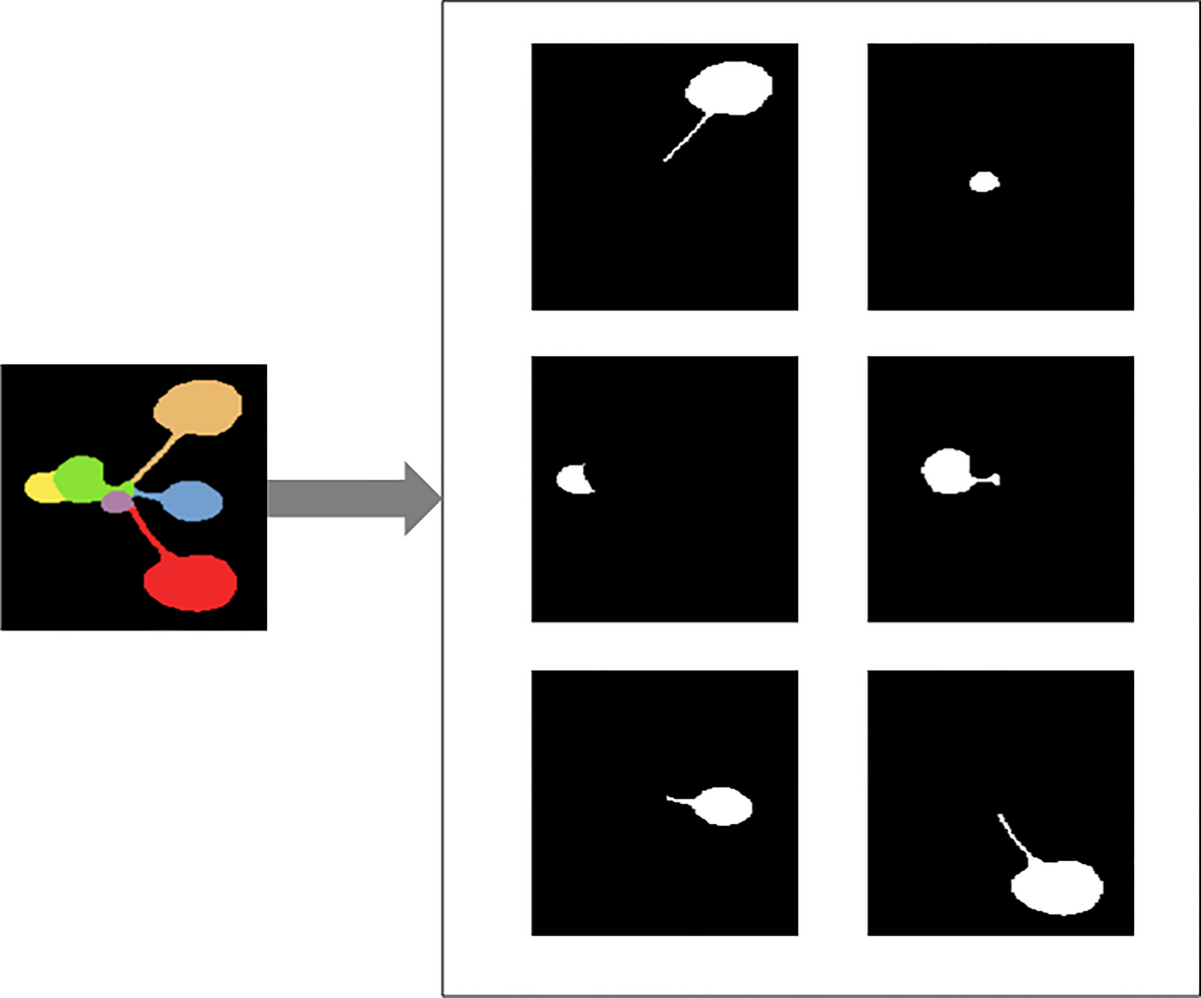
Illustration of converting color image of *Arabidopsis thaliana* to black and white images for each leaf.

In the first step, the RGB segmentation annotation image data set in CVPPP is converted into JSON files in Common Objects in Context(COCO) format. A separate black and white image of each leaf is generated from the color annotated leaf images provided in the dataset, and **Figure 3** gives an example of the completed conversion of a particular image, and a JavaScript Object Notation (JSON) file containing all the image information is generated.

In the second step, a tag file in XML format unique to each image is generated from the JSON file. In this tag file, the location of the image, the genotype, and the bounding box where each leaf is located are included, so all information such as the genotype, number of leaves, and leaf area of the image can be directly obtained through this tag file.

2. Data cleaning is mainly to remove irrelevant data and duplicate data from the original data set, smooth out noisy data, filter out data irrelevant to the mining topic, and deal with missing values, abnormal values, etc (Brownlee, 2020). Data cleaning in this paper focuses on comparing the number of leaves in each image obtained according to the data collection step with the number of leaves given in the original dataset and eliminating the parts with different numbers of leaves.

#### 3.2.2 Feature engineering

Feature engineering extracts features from the processed data in the data preparation phase and transforms them into formats that are suitable for the machine learning model (Zheng and Casari, 2018). It mainly includes three parts: feature selection, feature extraction, and feature construction. Feature selection reduces feature redundancy by selecting important features, feature extraction reduces the dimensionality of features by applying a specific mapping function, and feature construction extends the original feature space. In addition, it also includes feature improvement, feature dimensionality reduction, and other contents. Feature engineering maximizes the extraction of features for use in subsequent NAS processes.

### 3.3 Model architecture search

Neural architecture search is a sophisticated and systematic work, which is mainly based on the key components of NAS: search space, search strategy, and evaluation strategy (Ren et al., 2021).

Bayesian optimization(BO) (Shahriari et al., 2015) is an effective way for hyperparameter optimization, and has recently emerged as a very promising strategy for NAS. Bayesian optimization puts the optimization issue into a probabilistic framework by representing the agent function as a probability distribution, and then updating this distribution with new information. The acquisition function is used to evaluate the probability of obtaining a better result at a particular point in the exploitation space based on a known prior. The key to this is the balance between exploration and exploitation.

Auto-Keras (Jin et al., 2019) is guided by a Bayesian optimization algorithm to explore the search space by changing the neural structure. The range of fluctuations of the true target function values (i.e., mean and variance) is first estimated based on the function values of the already searched points, usually implemented with Gaussian process regression. Afterward, the acquisition function can be constructed from the mean and variance, i.e., an estimate of the probability that each point is the extreme point of the function, reflecting the degree to which each point is worth searching, and the extreme point of this function is the next search point. Finally, the newly sampled data is added to the set of observations and then recursive execution is performed until convergence or exhaustion of budgetary resources.

For the search algorithm, Auto-Keras uses *A* ^∗^ algorithm (Yao et al., 2010) for searching and simulated annealing, inspired by various heuristic search algorithms that explore tree-like search spaces and optimization methods that explore and exploit tradeoffs.

Whenever NAS generates a new neural network, it is first evaluated for performance. If the network is trained until convergence and then its performance is evaluated, it will consume a lot of time and computational resources. So the early stopping, low fidelity, surrogate, and parameter sharing skills are selected to speed up the evaluation. Auto-Keras uses network morphisms for the purpose of network weight parameter sharing. The sub-models share weights with each other, so there is no need to re-train the sub-models each time. It also uses the early stop method to stop the computation of configuration models that are expected to perform poorly on the validation set.

### 3.4 Execution process

**Figure 4:**
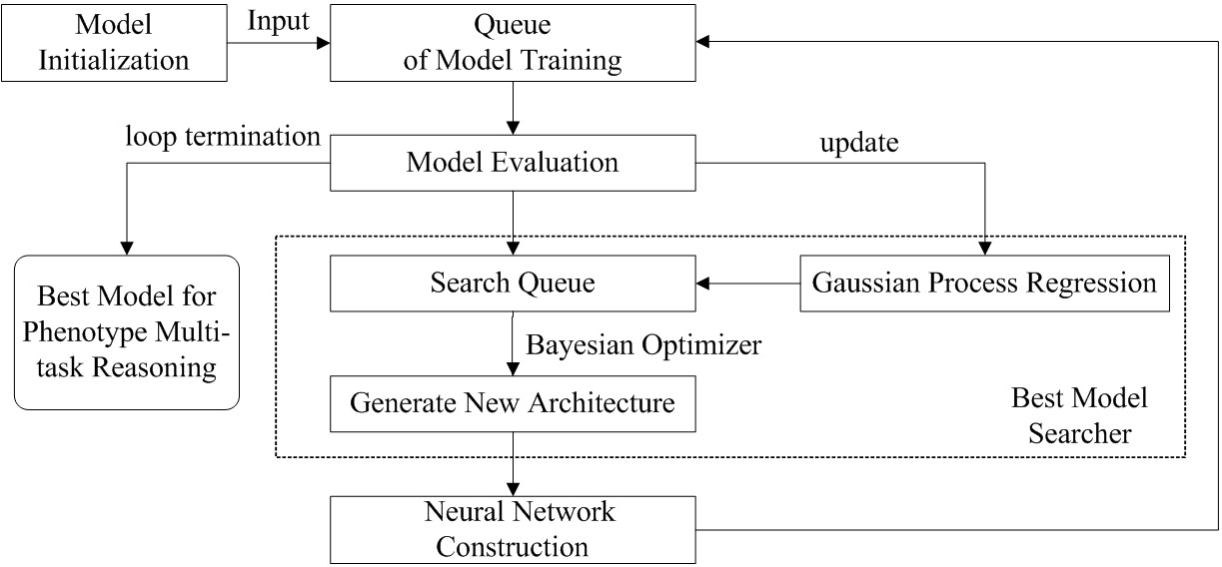
The parameters tuning and network structure selection process for *Arabidopsis thaliana* multi- task phenotype reasoning based on Auto-Keras.

In this paper, we construct amdeep AutoML model for *Arabidopsis thaliana* phenotype reasoning based on Auto-Keras (Jin et al., 2019). The execution process of automatic parameter tuning and network structure selection for generating the optimal model is shown in **Figure 4**.

There are 5 steps for optimizing multi-task phenotype reasoning:

(1) Put the network module into the generator as a seed to initialize the model. Three models including CNN, ResNet, and DenseNet can be selected. The number of initial network models constructed during initialization can be set by itself when calling the API;
(2) After the initialization, all the generated models will be put into the training queue, and the models from the queue head are taken out in turn for training;
(3) When training, the model is evaluated and put into the search queue, because the best model will eventually be trained again, so full training is not required at this time, and thus the neural network architecture search can be performed while training. The performance of the model is used as feedback to the Best Model Searcher to update the Gaussian Process;
(4) The model is removed from the search queue, the Bayesian Optimizer in the Searcher would generate a new architecture and the annealing algorithm is used to determine whether to perform network morphism. The following types of morphism are provided in Auto-Keras: depth, width, and connection between layers. Morphism is random, e.g. the choice of which morph is random, or when choosing to increase the width, the choice of which layer to widen is also random. If a new network architecture is generated by network morphism and that network architecture is not in the existing model, a Gaussian Process Regression is used to predict the better network structure, and the best-performing network is recorded and added to the model search queue and training queue;
(5) The search and training process in steps 3 and 4 is repeated continuously according to the predetermined number of network models to obtain the model with the best results.

After setting the number of training models, the number of iteration rounds, and the system resources available for training by Auto-Keras, the program will automatically adjust the model structure and each parameter according to the process shown in **Figure 4**, and using the visualization component, the final model structure after training can be obtained.

## 4 Experiments and analysis

### 4.1 Experimental environment

The experimental platform is Windows 10 with Intel(R) Xeon(R) Gold 5218 CPU, 32G RAM, GeForce RTX 2080 Ti GPU, and 11G video memory. Models are implemented with Tensorflow 2.0.0+, autokeras 1.0. 18, Python 3.7+, and CUDA 10.0+.

### 4.2 Dataset details

This experiment uses the Ara2013-Canon dataset (Minervini et al., 2016), a publicly available dataset obtained from the CVPPP leaf segmentation and counting challenge, for training. First, color segmentation of annotated images in CVPPP is used to generate JSON files in COCO format from the original data set. Then, the COCO format data set is converted to a VOC format data set, and the genotype, leaf count, and leaf area information of *Arabidopsis thaliana* are obtained directly from the XML label file.

There are five *Arabidopsis thaliana* genotypes in this dataset: col-0, ein2, ctr, adh1, and pgm. Each *Arabidopsis thaliana* image has label information of genotype, leaf number, leaf position box images, as shown in **Table 1**, with a total of 165 *Arabidopsis thaliana* phenotype images.

**Table 1:** Sample image of the Ara2013-Canon dataset.

Genotype	Col-0	ein2	pgm	ctr	adh 1
Original Image Leaf Segmentation Image	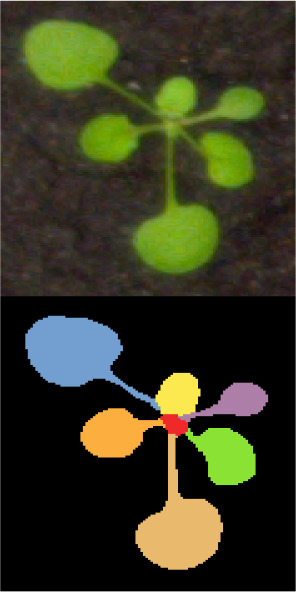	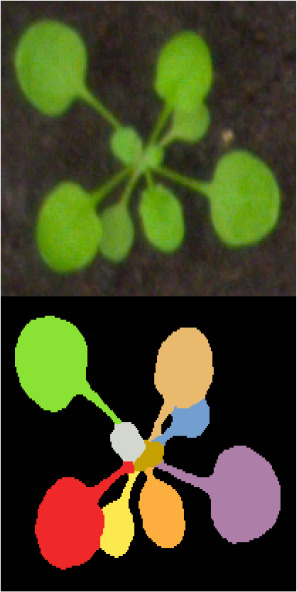	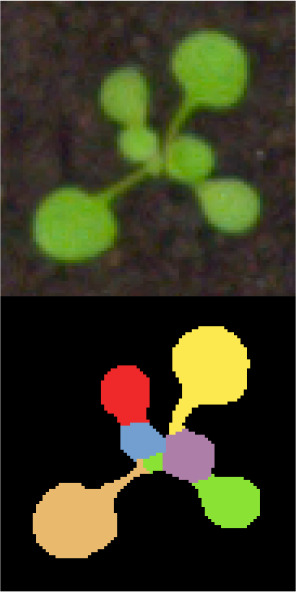	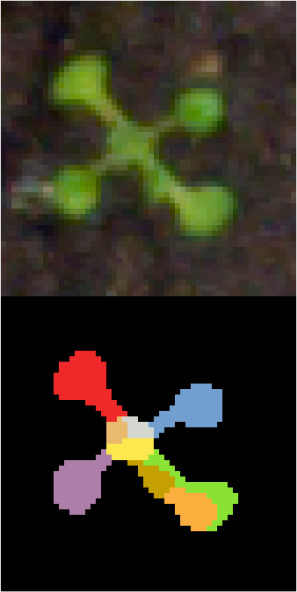	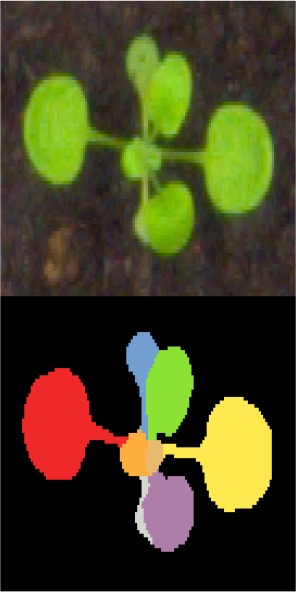

### 4.3 Evaluation metric

#### 4.3.1 Classification evaluation metrics

In classification tasks, Accuracy *A* is a frequently used evaluation metric, and it measures the proportion of correctly predicted samples to all samples. The formula is displayed in Equation 3. The prediction effect of the model is better in terms of accuracy the closer its value is near 1.


(3) $$A=\frac{TP+TN}{TP+TN+FP+FN}$$

where *TP* represents the positive sample predicted by the model as a positive class, *TN* represents the negative sample predicted by the model as a negative class, *FP* represents the negative sample predicted by the model as a positive class, and *FN* represents the positive sample predicted by the model as a negative class.

#### 4.3.2 Regression evaluation metrics

The reliability of the change in the dependent variable in the regression task is indicated by the coefficient of determination, *R* ^2^, which is defined as Equation 4. *R* ^2^ is a numerical feature used to illustrate the link between a random variable and many other random variables. The coefficient of determination might have a maximum value of 1. The regression line fits the predicted value better and becomes closer to the true value as the value gets closer to 1.


(4) $$R^{2}=\frac{\sum_{i=1}^{n}{\left({\hat{y}}_{i}-\bar{y}\right)}^{2}}{\sum_{i=1}^{n}{\left(y_{i}-\bar{y}\right)}^{2}}$$

The extreme errors caused by the squaring amplify the mean squared error(MSE), which is determined as the mean of the squared difference between the anticipated and actual observed values. Predicted values that deviate more from the genuine value are penalized more harshly than those that vary less. The prediction effect is more closely related to the true value the lower the MSE value, which is defined in Equation 5.


(5) $$MSE=\frac{1}{n}\sum_{i=1}^{n}{\left(y_{i}-{\hat{y}}_{i}\right)}^{2}$$

In the formula given above, *n* stands for the quantity of samples, y_i_ for the sample’s true value, ${\hat{y}}_{i}$ for its predicted value, and $\bar{y}$ for the average of the true values of all the samples.

### 4.4 Experimental results

#### 4.4.1 Auto network generation

A multi-task model for automated machine learning was built using Auto-Keras (Jin et al., 2019), setting the maximum number of trials to 10 and the number of iteration rounds to 300, and training the model with an input image of 28 × 28 × 3.

**Figure 5:**
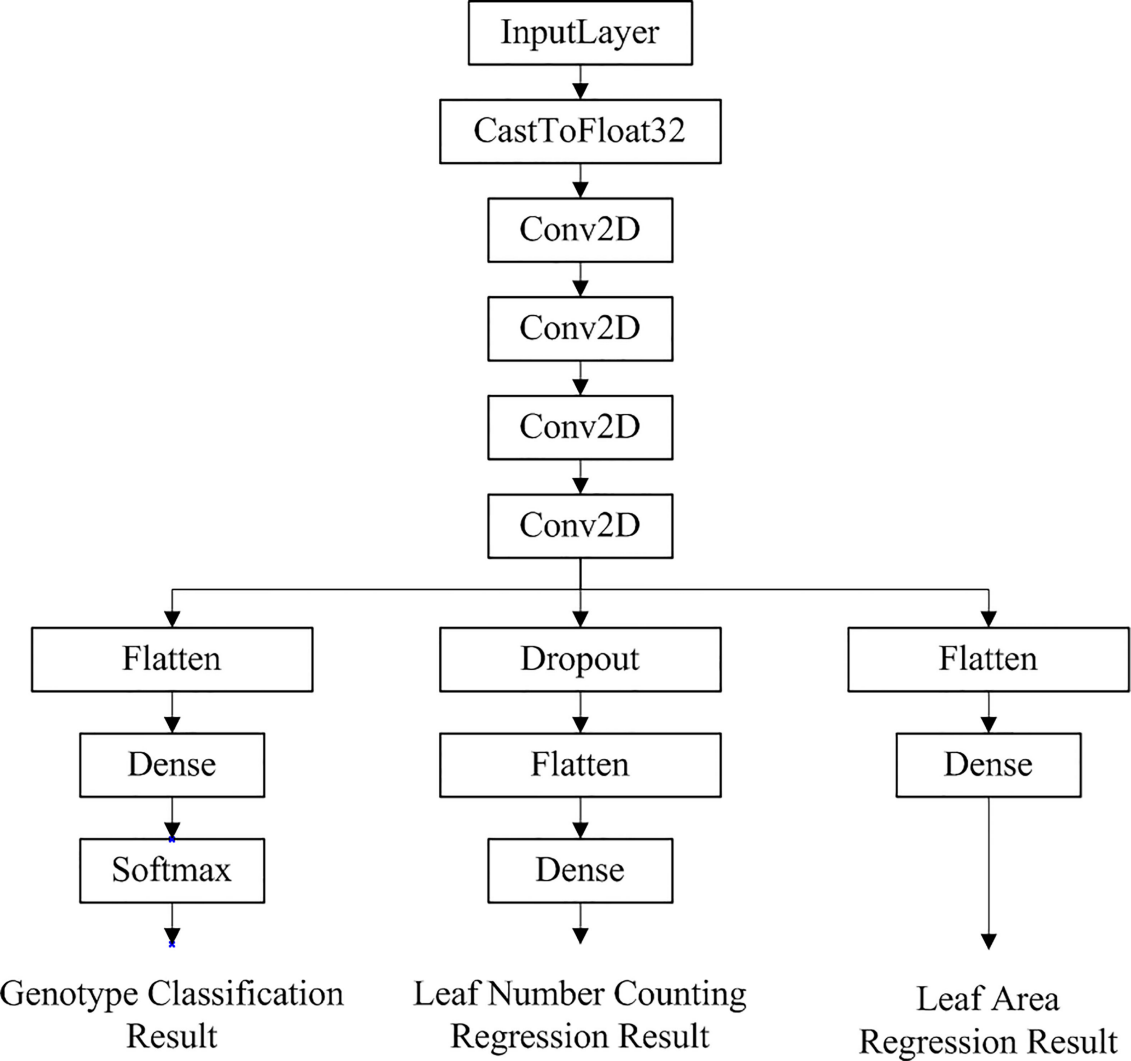
Network structure of the optimal multi-task learning model generated by Auto-Keras for *Arabidopsis thaliana* phenotype reasoning tasks.

Auto-Keras applies the Early Stop model evaluation criteria and does not fully train all the searched models. Thus only the best-performing models in the evaluation process will be fully trained. After training 10 models using the neural network architecture to search and automatically tune the models as well as the parameters, the model with the best predictions from the 10 trials was selected for the 11th full training. The final structure of the automated machine learning model generated by Auto-Keras’ model visualization tool for accomplishing multi-task learning is shown in **Figure 5**.

As can be seen in **Figure 5**, the multi-task learning model generated with the automated machine learning approach is a hard parameter sharing model, and the optimal network structure and parameters are automatically selected based on the neural network architecture search algorithm with flexible addition of network layers such as random deactivation. The total number of parameters that have been trained for the model is 118,247, and the specific parameters are shown in **Table 2**.

**Table 2:** Network parameters of the trained and selected model shown in **Figure 5** generated by AutoML for *Arabidopsis thaliana* phenotype reasoning.

No.	Layer	Output Shape	Parameters	Connected to
1	input_1	(None,28,28,3)	0	–
2	cast_to_float32	(None,28,28,3)	0	input_1
3	conv2d	(None,26,26,32)	896	cast_to_float32
4	conv2d_1	(None,24,24,32)	9248	con2d
5	conv2d_2	(None,22,22,32)	9248	con2d_1
6	conv2d_3	(None,20,20,32)	9248	con2d_2
7	flatten	(None,12800)	0	con2d_3
8	dropout	(None,20,20,32)	0	con2d_3
9	dense	(None,5)	64005	flatten
10	flatten_1	(None,12800)	0	dropout
11	flatten_2	(None,12800)	0	cov2d_3
12	classification_head_1	(None,5)	0	dense
13	regression_head_1	(None,1)	12801	flatten_1
14	regression_head_2	(None,1)	12801	flatten_2

#### 4.4.2 Training loss

**Figure 6:**
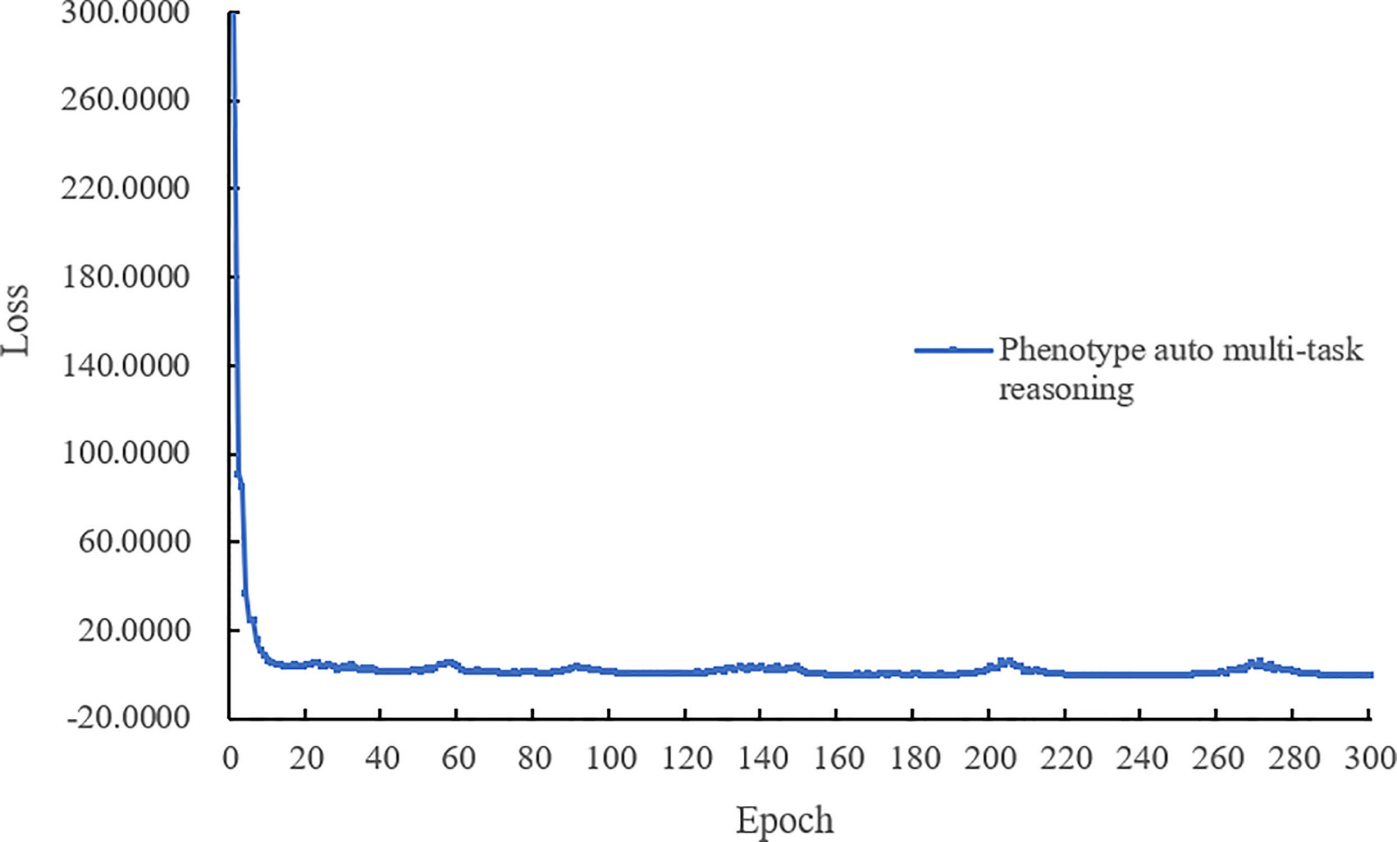
Total loss curve of the optimal model during training.

The last complete model training process of the loss value curve is shown in **Figure 6**, the loss decreases quickly with epoch, and when the epoch is lower than 15. **Figure 6** shows that our model performs well during training without oscillation and can eventually reach convergence.

#### 4.4.3 Results reasoning and comparison

**Figure 7:**
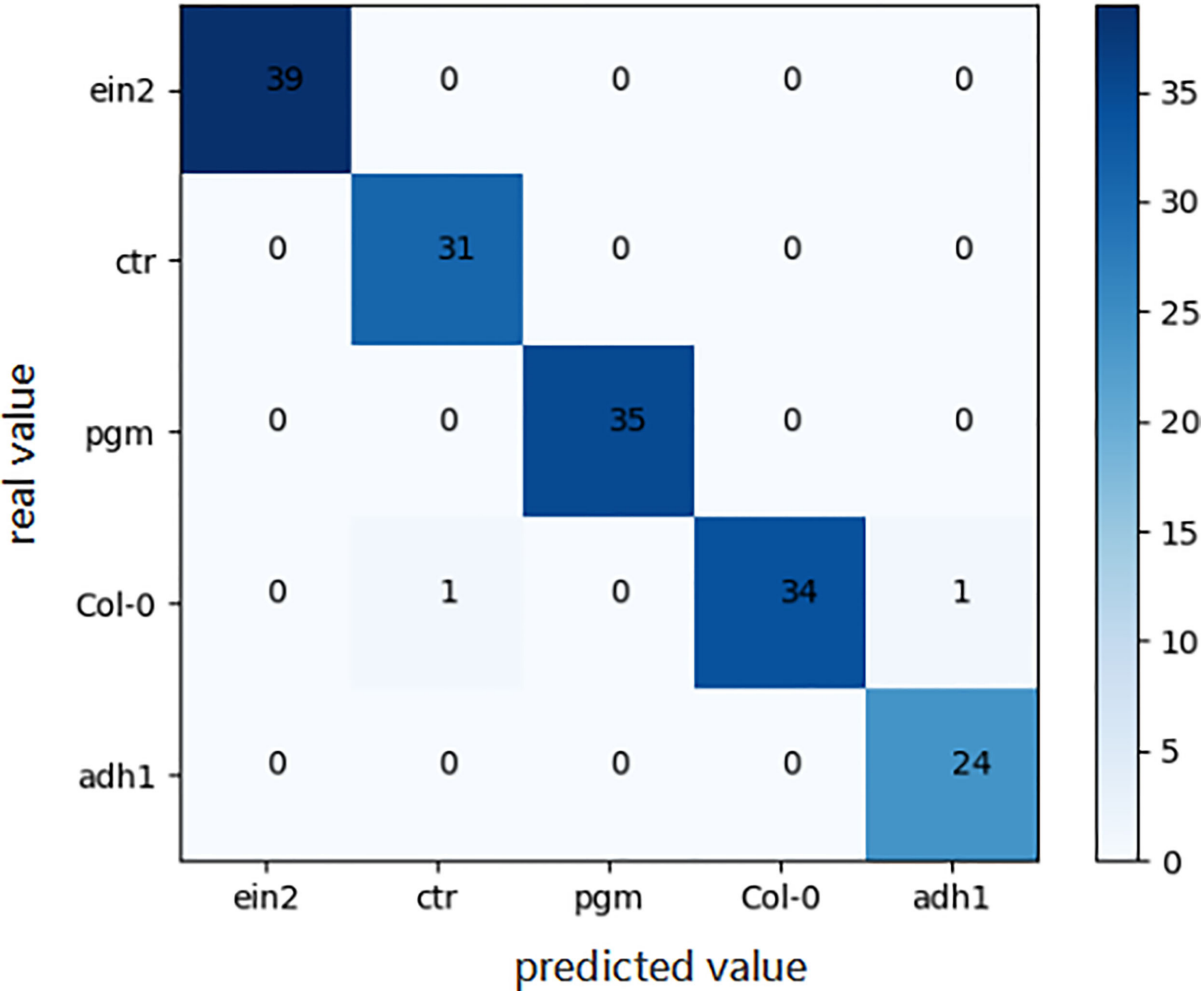
Confusion matrix of the optimal model on genotype classification task.

For the genotype classification task, the confusion matrix of the final model obtained by the aforementioned automated machine learning approach is shown in **Figure 7**. As shown in **Figure 7**, only two *Arabidopsis thaliana* samples belonging to Col0 type were mistakenly classified as other genotypes, which indicates the great accuracy of our model in the task of genotype classification.

**Figure 8:**
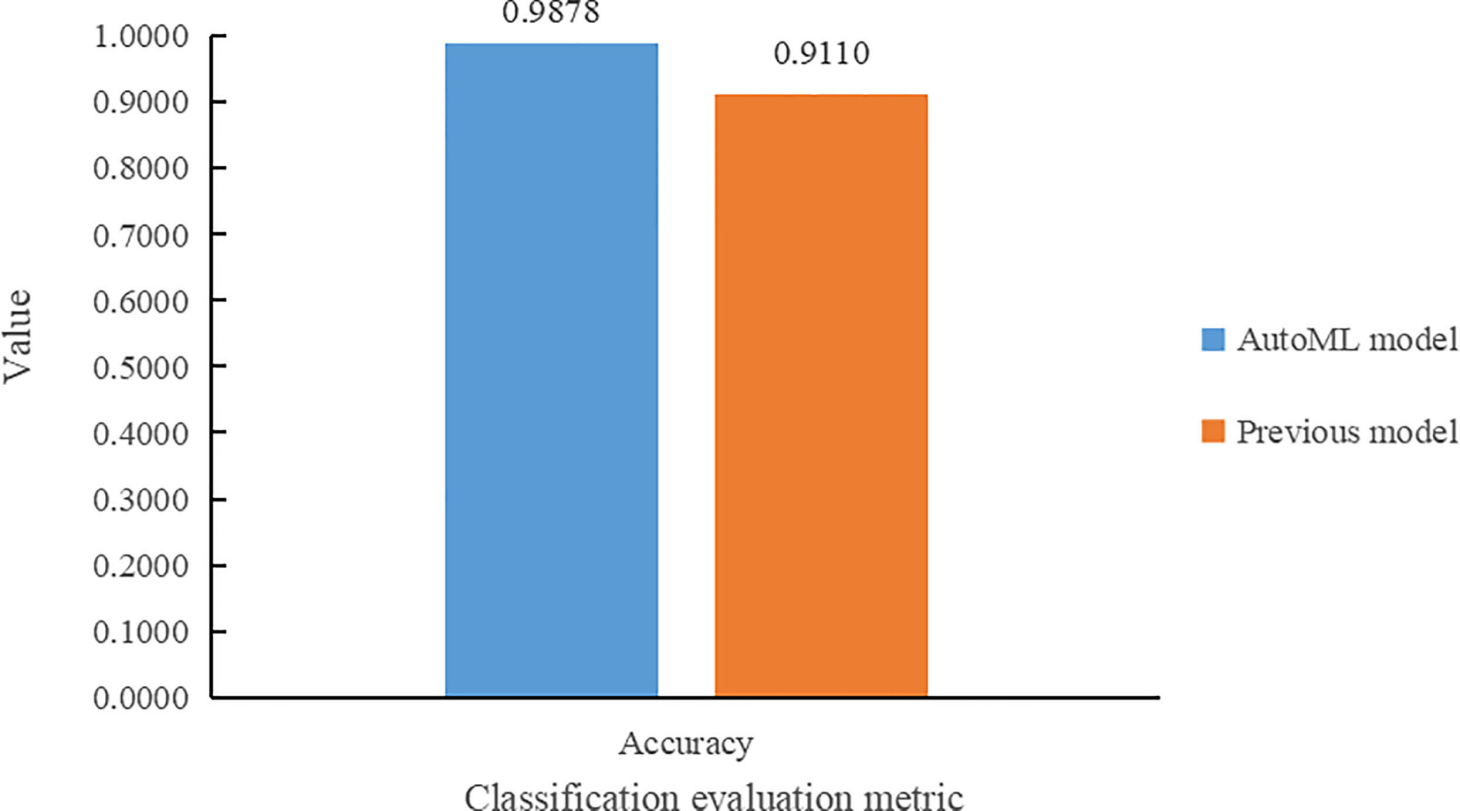
Comparative histogram of the two models in terms of classification accuracy.

Results comparison with Dobrescu et al. (2020) of the in terms of classification accuracy metrics is shown in **Figure 8**. From **Figure 8**, it can be seen that the model trained using the auto multi-task outperforms the model of Dobrescu et al. (2020) in the genotype classification task, with an improvement in classification accuracy of 7.68%.

**Figure 9:**
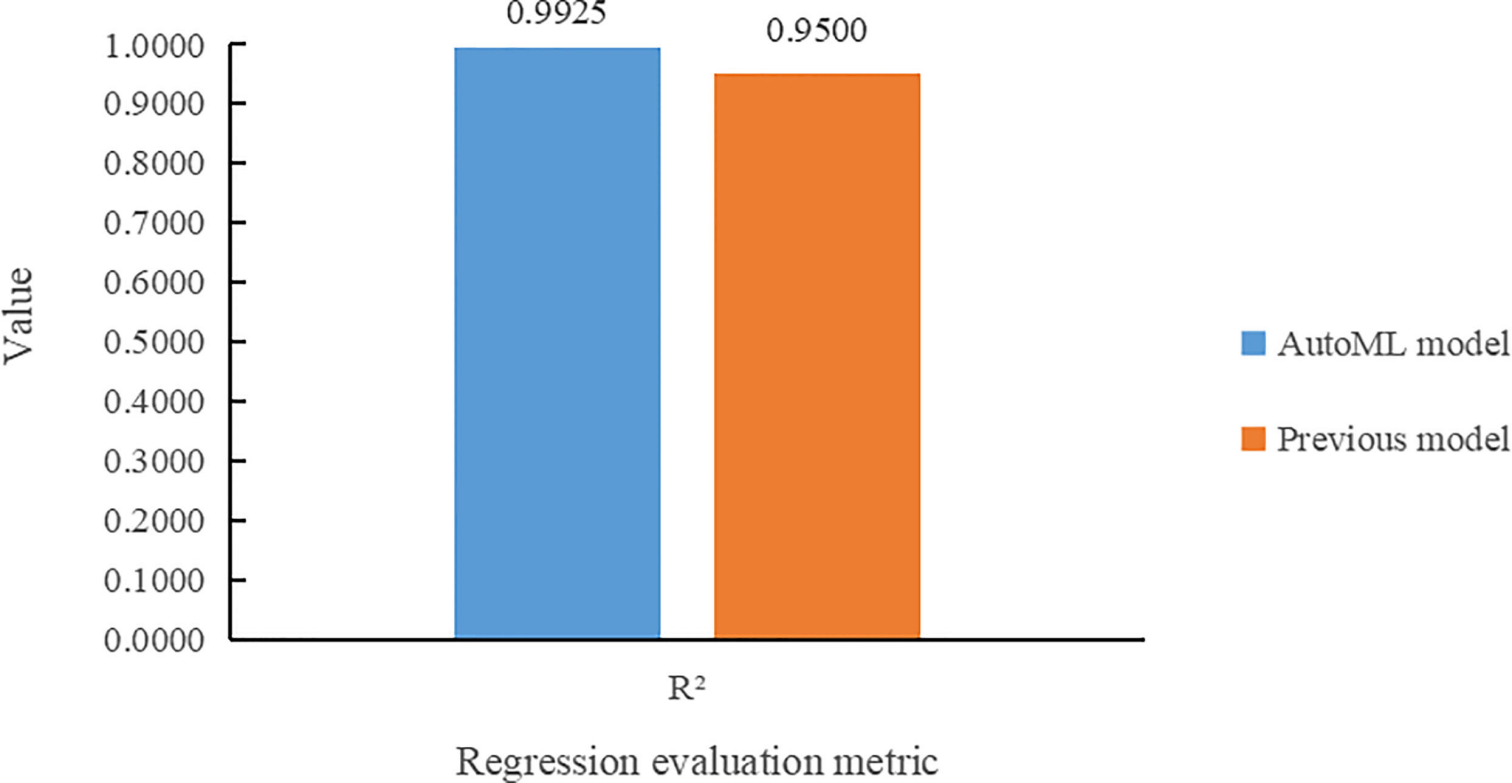
Comparative histogram of *R* ^2^ for the two models in the leaf number regression task.

**Figure 9** compares the two models according to *R* ^2^ on the task of leaf number regression. It can be seen that the multi-task learning model built by the AutoML not only makes more accurate prediction of the leaf number, but also improves its *R* ^2^ value by 4.25% over the previous model.

**Figure 10:**
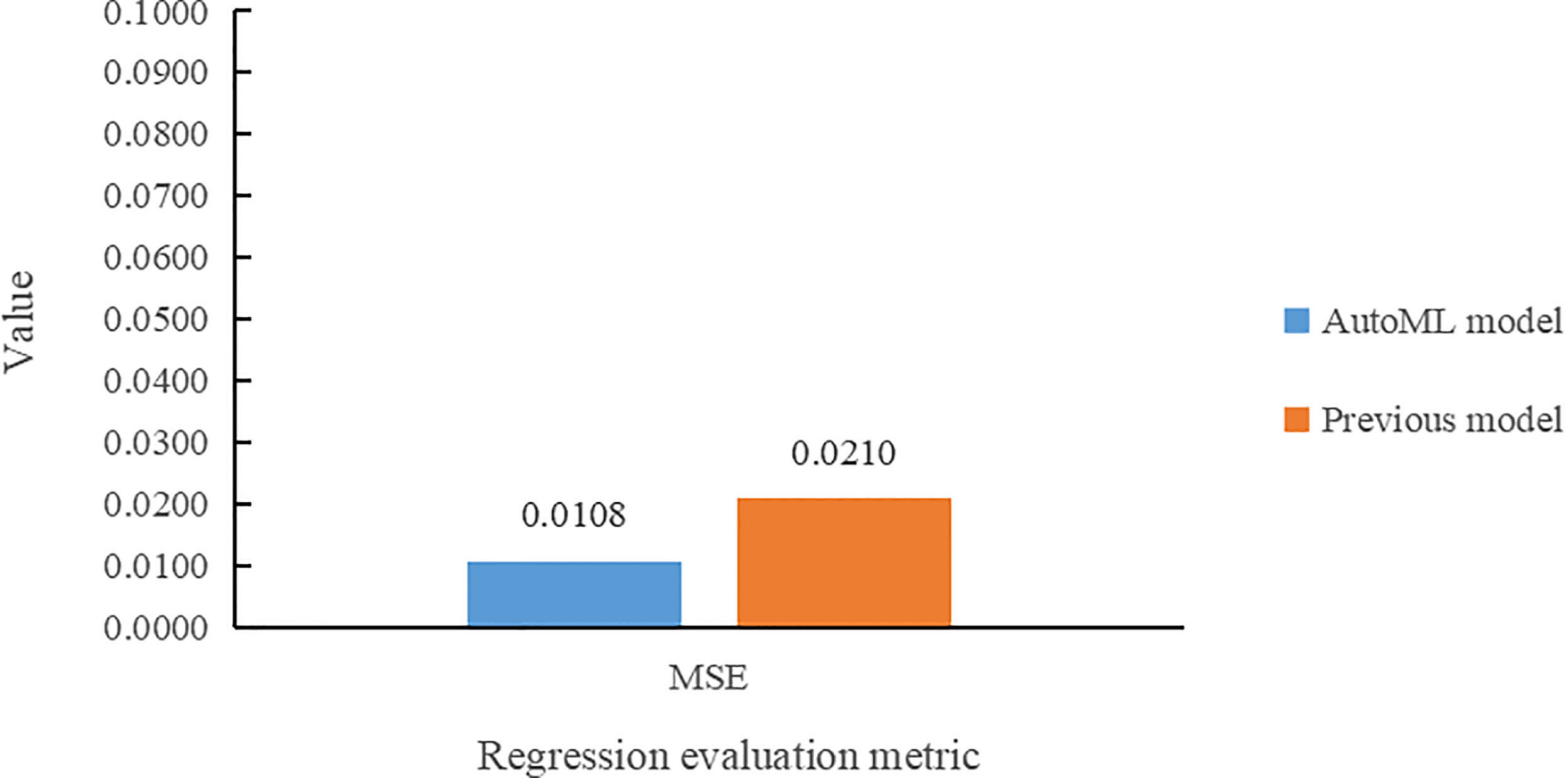
Comparative histogram of MSE for the two models in the leaf area regression task.

In the leaf area regression task, the comparison of the two models in terms of MSE value is presented in **Figure 10**. The multi-task learning model developed using AutoML had the smallest MSE error value for leaf area prediction and also reduced by 1.02% compared to the previous model.

Combining the experimental results of the two tasks, it can be seen that automated machine learning can automatically adjust the structure and hyperparameters of the model to obtain a higher model classification accuracy and a lower prediction error without a lot of human intervention. It not only makes up for the shortcomings of manually constructed models, but also improves classification accuracy and prediction accuracy compared with the previous model.

Although it takes longer to train the model than other methods, the stochastic nature of model construction makes it difficult for the best model obtained to be reproduced by others. Automated machine learning facilitates the model building process and can be used by a wide range of people, making it easy for almost anyone to build a model suitable for their task, and allowing experts and scholars to focus their research on more important goals rather than spending a lot of time in tweaking the model.

### 4.5 Complexity discussion

For the model training of the *Arabidopsis thaliana* phenotype reasoning system, the time complexity depends primarily on NAS as *O*(*nt* + *t_best_*), where *n* is the number of network architectures to be searched, and *t* is the average training time for all networks. Auto-Keras reduces *t* by generating a new network structure on top of the original network through network morphism, which allows the new network to perform better with fewer iterations. And *t_best_* is the time for one complete training of the final selected optimal model. When the model training is completed, our system simply feeds the image to be analyzed into the already trained model to obtain the information on genotype, leaf number, and leaf area with time complexity of *O*(1). Thus, the time complexity of the system depends on the efficiency of the NAS and the size of the search space.

Therefore, we can draw the conclusion that the strength of using AutoML to construct multi-task learning for *Arabidopsis thaliana* phenotype reasoning not only considering the correlation between tasks, but also achieves a joint improvement of multiple objectives of tasks through parameter sharing and joint training. Furthermore, it also takes advantage of the points of automatic machine learning to select the best models and adjust hyperparameters tuning, and finally obtain better performance.

## 5 System implementation and deployment

### 5.1 System workflow

**Figure 11:**
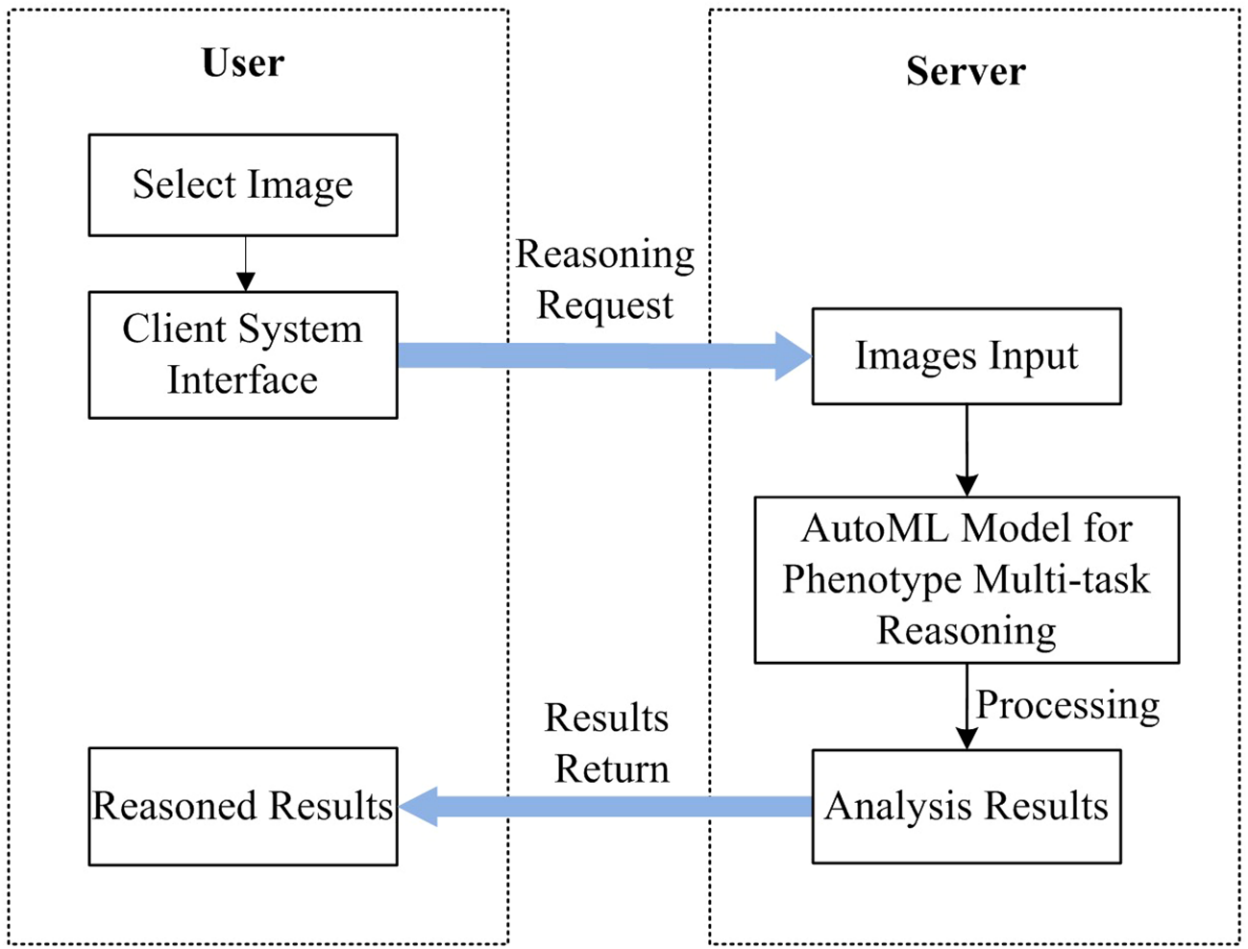
Workflow of *Arabidopsis thaliana* phenotype reasoning system based on AutoML.

The system includes two components of the user and the server, such as AWS of Amazon, or Alibaba Cloud, which can be deployed on the cloud. The server deploys the trained AutoML multi-task model and leverages GPU or CPU for inference while the user primarily handles the action of picking the recognized images for the user. **Figure 11** depicts the unique workflow when using the system to analyze plant photos.

As shown in **Figure 11**, There are 5 steps in all: 1) A recognition request is submitted after the user chooses the image to be recognized using the system at their end; 2) The front-end system sends the back-end server the data it has been asked for; 3) Based on the requested input, the server reads in photos and feeds them into a model that has been trained using the AutoML approach; 4) Obtain the expected data following the conclusion of the model processing and deliver the analysis findings; and 5) The user interface shows the image processing outcomes so that users may see the data graphically.

### 5.2 Main functions

**Figure 12:**
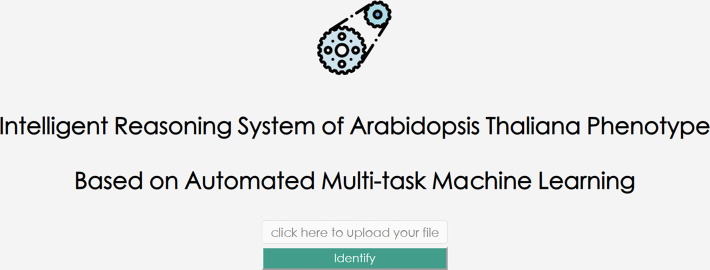
User’s client of *Arabidopsis thaliana* phenotype reasoning system.

Based on its server’s URL address, the server will generate a hyperlink address for users to access. The home page of the online *Arabidopsis thaliana* phenotypic reasoning system based on AutoML can be accessed by this address, as shown in **Figure 12**.

**Figure 13:**
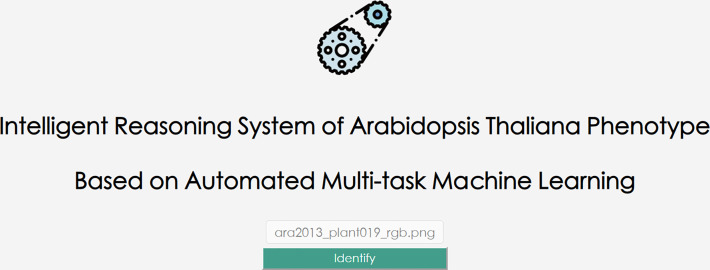
Upload *Arabidopsis thaliana* phenotype image for multi-task reasoning.

After choosing the image that needs to be processed and analyzed, click “click here to upload your file” in the main interface. The image’s file name will then be presented in the main interface, as shown in **Figure 13**.

**Figure 14:**
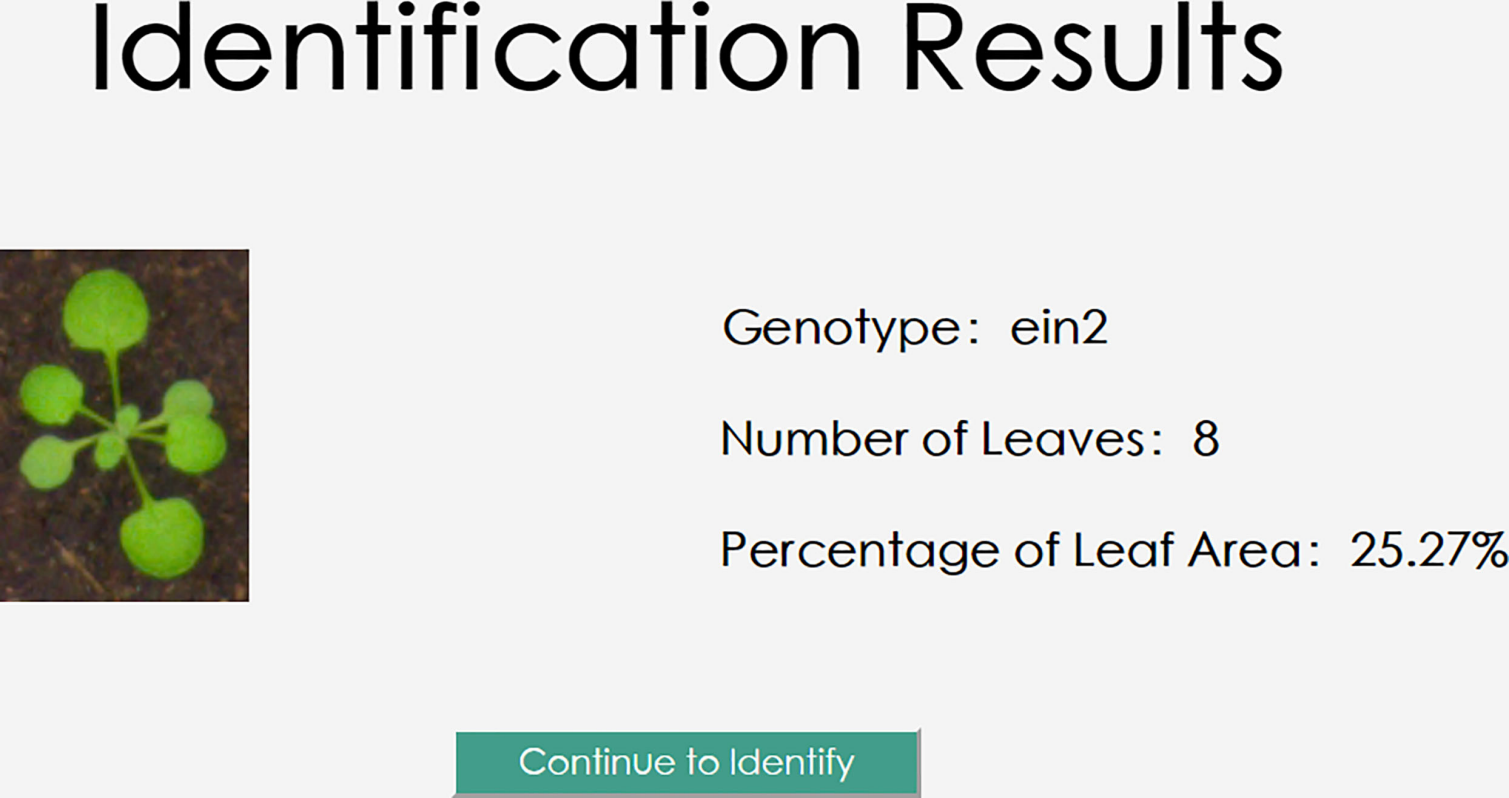
Illustration of *Arabidopsis thaliana* phenotype multi-task reasoning results.

The user will create the request data and send it to the back-end server by clicking the “Identify” button. To perform inference and retrieve the *Arabidopsis thaliana* results of genotype classification, leaf number regression, and leaf area regression, the server will read in the images and input them into the trained model based on the request *Arabidopsis thaliana* data. The outcomes of the three jobs will be returned to the user client by the model on the server, and these will be illustrated on the user client as illustrated in **Figure 14**.

## 6 Conclusions and future work

In this paper, we propose AutoML based multi-task intelligent reasoning system for the *Arabidopsis thaliana* phenotype. Our method can select the best model and perform parameter tuning automatically for multi-task learning for *Arabidopsis thaliana* phenotype analysis. The optimal genotypic classification, leaf number, and leaf area prediction results of the present *Arabidopsis thaliana* data set were obtained by training the multi-task learning model with AutoML. The conclusions are summarized as the following.

(1) The multi-task learning model trained by AutoML of Auto-Keras achieved 98.78% accuracy in *Arabidopsis thaliana* genotype classification task, and 7.68% higher than Dobrescu’s model. In the leaf counting regression task, the value of *R* ^2^ is 0.9925, and 4.25% higher than the previous model. In leaf area regression task, the MSE value is 0.0108, which is 1.02% lower than Dobrescu’s work (Dobrescu et al., 2020).
(2) Our method can train and adjust model structure and parameter tuning automatically for plant phenotype multi-task reasoning, and improve the classification and regression ability of models automatically without human intervention.

The dataset used in this paper is relatively small and can be expanded in subsequent studies. In future research, various different AutoML frameworks can be used to build the model and compare which method can obtain better overall performance with this dataset. More phenotypic classification or regression tasks can be added to the multitask learning model, and a system dedicated to analyzing plant phenotypes can be built, which can be extended to other plant phenotypic studies.

## Data availability statement

The raw data supporting the conclusions of this article will be made available by the authors, without undue reservation.

## Author contributions

PY: Conceptualization, Methodology, Reviewing, Supervision. SX: Data analysis, Writing Original draft,Software. ZZ: Reviewing and Editing. HX: Reviewing, Supervision. All authors contributed to the article and approved the submitted version
